# Semi-Supervised Domain Adaptation for Holistic Counting under Label Gap

**DOI:** 10.3390/jimaging7100198

**Published:** 2021-09-29

**Authors:** Mattia Litrico, Sebastiano Battiato, Sotirios A. Tsaftaris, Mario Valerio Giuffrida

**Affiliations:** 1 Department of Mathematics and Computer Science, University of Catania, 95125 Catania, Italy; mattia.litrico@studium.unict.it (M.L.); battiato@dmi.unict.it (S.B.); 2 School of Engineering, University of Edinburgh, Edinburgh EH9 3FG, UK; s.tsaftaris@ed.ac.uk; 3 School of Computing, Edinburgh Napier University, Edinburgh EH10 5DT, UK

**Keywords:** domain adaptation, holistic counting, regression, label gap

## Abstract

This paper proposes a novel approach for semi-supervised domain adaptation for holistic regression tasks, where a DNN predicts a continuous value y∈R given an input image *x*. The current literature generally lacks specific domain adaptation approaches for this task, as most of them mostly focus on classification. In the context of holistic regression, most of the real-world datasets not only exhibit a covariate (or domain) shift, but also a label gap—the target dataset may contain labels not included in the source dataset (and vice versa). We propose an approach tackling both covariate and label gap in a unified training framework. Specifically, a Generative Adversarial Network (GAN) is used to reduce covariate shift, and label gap is mitigated via label normalisation. To avoid overfitting, we propose a stopping criterion that simultaneously takes advantage of the Maximum Mean Discrepancy and the GAN Global Optimality condition. To restore the original label range—that was previously normalised—a handful of annotated images from the target domain are used. Our experimental results, run on 3 different datasets, demonstrate that our approach drastically outperforms the state-of-the-art across the board. Specifically, for the cell counting problem, the mean squared error (MSE) is reduced from 759 to 5.62; in the case of the pedestrian dataset, our approach lowered the MSE from 131 to 1.47. For the last experimental setup, we borrowed a task from plant biology, i.e., counting the number of leaves in a plant, and we ran two series of experiments, showing the MSE is reduced from 2.36 to 0.88 (intra-species), and from 1.48 to 0.6 (inter-species).

## 1. Introduction

According to [1], domain adaptation methods can be classified based on the relation between the label sets of the source and target domains. Let $Y_{S}$ and $Y_{T}$ be the label sets for the source and target domains, domain adaptation algorithms can be classified as: *closed set* ($Y_{S}=Y_{T}$), *open set* ($Y_{S}\cap Y_{T}\neq \emptyset$), *partial* ($Y_{T}\subset Y_{S}$), and *universal* (no prior knowledge of the label sets is available). Domain Adaptation (DA) is a machine learning task that transfers a trained model f(x) to a new (and unseen) dataset. In particular, when a model $f(x_{s})$ is trained on a (source) dataset $X_{S}$ to perform a task T, we want the same model to also generalise on a different (target) dataset $X_{T}$. Generally speaking, domain adaptation is challenged by covariate (or domain) shift: the marginal distributions of source $D_{S}$ and target $D_{T}$ datasets are different, i.e., $D_{S}\neq D_{T}$ [2].

**Figure 1:**
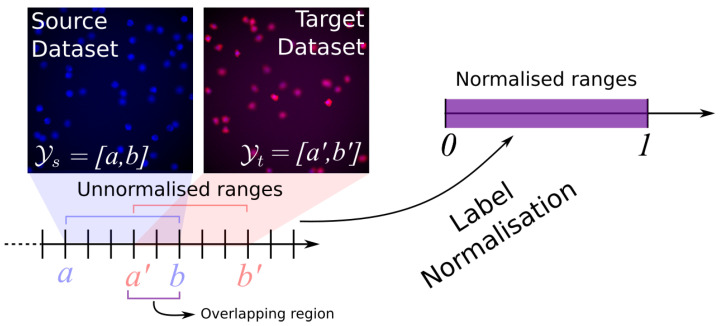
Source and Target datasets have two different label ranges ([a,b] and $\left[a^{\prime },b^{\prime }\right]$ respectively), causing a label gap. Although overlaps may occur (as shown), the label gap challenges most of the state-of-the-art solutions for regression problems. Our proposed solution is to perform label normalisation, i.e., scaling source (and target) labels into [0,1].

To minimise the covariate shift, several approaches have been proposed, such as Maximum Mean Discrepancy (MMD) [3], adversarial training [4,5,6,7], as well as style-transfer [8]. DA has recently been mostly investigated for classification tasks, showing outstanding results on closed set [5,7,9,10,11], open set [12,13], partial [14,15], and even universal cases [16]. However, regression tasks have attracted less attention in the computer vision community. In particular, in this paper we investigate DA for holistic regression. Specifically, we want to have a model f(x)↦y, with y∈Y⊆R, i.e., given an image x∈X as an input to f(x), the model predicts a continuous value. Examples of holistic regression applications are counting [17,18], age estimation [19], and time series forecasting [20]. Domain adaptation for holistic regression is more prone to *label gap*, i.e., the target dataset may contain values that are not contained in the source dataset (in [16], this is referred to as *category gap*; we use the term label gap to be more generic to accommodate our application). This phenomenon is depicted in Figure 1.

This paper answers the question: *can we perform DA when the predicted variable is continuous, under label gap?* Inspired by [7,18], we propose a novel semi-supervised DA technique that transfers the model’s knowledge (from a source to a target dataset) in the holistic regression context. Specifically, we minimise covariate shift using adversarial training to align source and target image representations. We tackle the label gap by normalising the range of the source labels $Y_{S}=\left[a,b\right]$ into the range [0,1], as shown in Figure 1. As the network learns to predict numbers in the normalised range [0,1], we fine-tune the final layers of the network (i.e., the ones responsible for learning the regression task) with a handful of random annotated images sampled from $X_{T}$ (this is the only semi-supervised step of our method), to make predictions in the target dataset in the set of labels $Y_{T}=\left[a^{\prime },b^{\prime }\right]$. To avoid overfitting, we propose a stopping criterion that takes advantage of both MMD [21] and GAN Global Optimality Condition [22]. Instead of setting a maximum number of iterations, we jointly monitor the discrepancy between source and target, and the expected output of the discriminator.

We evaluate our method in 3 different scenarios (one synthetic, two real-world applications): cell, pedestrian, and leaf counting. The experimental results show that our method outperforms DANN [5] and the approach in [18] across the board. In particular, leaf counting experiments show the robustness of our method in the case of limited training data (both source and target domains have less than 1000 samples).

### Contribution

- We propose a semi-supervised domain adaptation method for holistic regression tasks that jointly tackles covariate shift and label gap.
- Label gap is mitigated via label normalisation, i.e., [a,b]↦[0,1]. As a consequence, our method works under *closed set*, *open set*, and *partial* DA [1].
- We demonstrate that as few as 10 annotated images taken from the target dataset are enough to restore the target label range, i.e., remapping $\left[0,1\right]\mapsto \left[a^{\prime },b^{\prime }\right]$.
- We propose a stopping criterion that jointly monitors the MMD and the GAN Global Optimality Condition to prevent overfitting and, thus, to better align source and target features. We show the effectiveness of this stopping criterion with an ablation study.

## 2. Related Works

In this section, we firstly discuss the related works on (unsupervised) domain adaptation. Then, we illustrate domain adaptation approaches on regression tasks. Lastly, we discuss the label gap problem. All the utilised mathematical notation is detailed in the Supplementary Materials.

### 2.1. Domain Adaptation

Several DA approaches have been proposed for different visual tasks, such as object recognition [23], face recognition [24], and image segmentation [25]. Formally, given a source domain $X_{S}$ and a trained model $f(x_{s})$ on the dataset $x_{s}\in X_{S}$ to solve a specific task T, we aim to generalise f(·) on a new unseen target dataset $X_{T}$. The case when the target labels $Y_{T}$ are not provided is called *unsupervised DA* (UDA).

The typical approach to (unsupervised) DA is to minimise the distance between the source and target feature space (covariate shift). Let $\phi_{S}\left(\cdot \right)$ be a feature extractor for the source domain, $\Phi_{S}$ be the source representation space, $\Phi_{S}=\left\{\phi_{S}\left(x\right)\;|\;x\in X_{S}\right\}$, and let $\Phi_{T}$ be the target representation space, the goal is to minimise the function:

$$\min_{\Phi_{S},\Phi_{T}}d\left(\Phi_{S},\Phi_{T}\right),$$

where d(·;·) is any (differentiable) distance function. Different choices of d(·;·) lead to different methodologies. In [3], the authors proposed a Deep Adaptation Network (DAN) that minimises Maximum Mean Discrepancy (MMD) as distance function. In [26], the authors proposed the Correlation Alignment (CORAL) loss to minimise the domain discrepancy. Then, ref. [7] proposed the Adversarial Discriminative Domain Adaptation (ADDA), using adversarial learning to reduce the covariate shift. In [5], the authors proposed the Domain-Adversarial Neural Network (DANN) that integrates a gradient reversal layer into the network to promote the extraction of features that are discriminative for the main learning tasks, whilst are indiscriminative for domain classification. The key idea of both ADDA and DANN is that, if the model is unable to recognise the domain from a set of features, then the domain shift has been minimised. Other adversarial learning approaches have been proposed in [6,15]. For example, in [27] the authors proposed to reduce the covariate shift with a per-batch feature whitening approach, mapping source and target features in a common (spherical) representation space. This approach inspired us to tackle the label gap instead, by label normalisation. Differently than others, our solution also does not require any assumption about the labels from both source and target datasets. In fact, several proposed DA approaches make some assumptions about the label sets (e.g., closed set, open set or partial domain adaptation) [3,5,7,10,12,13,14,15,26]. On the contrary, new *universal* approaches have been recently proposed [16,28], where such assumptions are not required. Although our method does not make such assumptions, we do not use the term universal, as we require a small labelled portion (semi-supervised) of the target dataset to map the predictions to the range of values $Y_{T}$.

### 2.2. Domain Adaptation for Regression Tasks

All the aforementioned approaches are typically focused on classification tasks, with less emphasis on (holistic) regression. In [29], the authors proposed an unsupervised DA method to estimate the Cardiothoracic Ratio, by predicting the segmentation masks of chest organs from X-rays images. In [30], the authors proposed a DA method to estimate crowd counting using density map predictions. Most of the recent counting approaches predict density maps and, although they also provide spatial information about the location of the counted objects, they are typically challenged by the scale variation [31].

However, some related works cast the counting problem as a holistic regression task, where the interest is to estimate the total number of objects regardless of their position. As such, a recent UDA for regression tasks has been proposed. Specifically, in [18], the authors built upon ADDA [7] to perform DA on a plant biology application (leaf counting). Although promising, this approach is challenged by the label gap, as demonstrated in our results. This is because the ranges of $Y_{S}$ and $Y_{T}$ are hardly the same in real applications.

### 2.3. Label Gap

Most of the DA approaches assume identical label sets (closed set) between domains. However, this assumption does not hold in many realistic scenarios, such as counting. In the last years, some works have been proposed to work on situation of open set [12,13], partial DA [14,15] or universal DA [16] for classification tasks, but they are not devised to work on continuous label space. For instance, the approaches in [12,13,16] add a new *unknown* class in order to face the label gap problem. Therefore, images in the target domain that belong to classes that are not in common across source and target domains are identified and, then, assigned to the unknown class. Obviously in the holistic counting task, this strategy cannot be used and the network has to be able to predict values even on target images that have labels not included in the source label set $Y_{S}$. At the same time, the approaches in [32,33] have been devised to work on regression tasks but they only work in situation of, respectively, partial DA and target shift.

To the best of our knowledge there is no state-of-the-art algorithm for holistic regression that does not require any assumption about the relationship between the label sets of source and target domains.

## 3. Proposed Method

**Figure 2:**
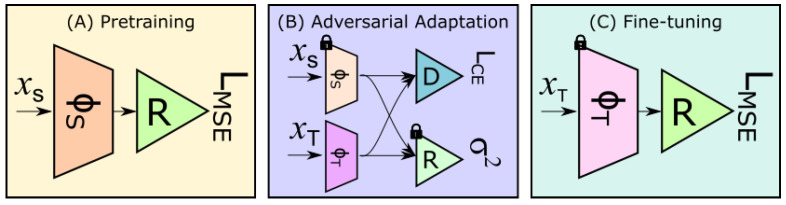
Overview of our training approach: $\phi_{S}$ is the feature extractor for the source dataset ($\phi_{T}$ for the target dataset); *R* is the regressor network; *D* is the discriminator; $L_{MSE}$ indicates the mean square error loss function ($L_{CE}$ is the cross-entropy loss); $\sigma^{2}$ is the variance-based regulariser preventing posterior collapse; locks indicate networks with fixed weights. The model obtained from the fine-tuning step is used for inference. (Best viewed in colour.)

We build our method upon  [7,18] and the training pipeline can be divided into three steps (cf. Figure 2): (i) pre-training; (ii) adversarial adaptation; (iii) semi-supervised fine-tuning of the regressor network. Our architecture includes the following blocks:

**Features Extractor**ϕ**:** We used ResNet-50 [34] as feature extractor that outputs a vector of size 2048.
**Regressor Network**R**:** It stacks 3 fully-connected (cf. Table 1) to learn the holistic regression task.
**Generator:** As in [7,18], the Feature Extractor acts as (feature) generator during the adversarial training to minimise the covariate shift.
**Discriminator**D: The architecture of the discriminator is also detailed in Table 1. *D* is trained such that it cannot differentiate between source and target features.

### 3.1. Pretraining on the Source Dataset

As shown in Figure 2A, this step pretrains both the $\phi_{S}$ and the regressor network *R* on the source dataset $X_{S}$ in a supervised manner with a *mean square error* (MSE) loss. To tackle the label gap, we normalise the labels $Y_{S}$ from [a,b] to [0,1] by replacing each label $y_{s}\leftarrow \frac{y_{s}-a}{b-a}$. This operation also helps to tackle the label gap between source and target datasets.

### 3.2. Feature Alignment with Adversarial Adaptation

For this step, we add the feature extractor for the target dataset $\phi_{T}$ (initialised with the weights obtained in the previous step), and the discriminator *D* for the adversarial training, as in [18]. An overview of this network is displayed in Figure 2B. In this step, the weights of $\phi_{S}$ and *R* are fixed. During training, $\phi_{T}$ acts as a generator of *fake* image representations, while $\phi_{S}$ outputs *real* features. In this way, $\phi_{T}$ is trained to generate features as similar as the ones produced by $\phi_{S}$, i.e., $D_{S}\approx D_{T}$.

To train an adversarial network, any *f*-divergence loss function can be used [35]. For instance, in [18], the authors used two different loss functions, i.e., cross-entropy [22] and least square [36], as one worked better in a different setup than the other. Here, we use the cross entropy as loss function for two reasons: (i) as demonstrated in Section 4, our approach works well across different scenarios (e.g., we do not need different losses for each scenario); (ii) we exploit the GAN Global Optimality condition as part of the proposed stopping criterion.

Hence, the generator $\phi_{T}$ and the discriminator are alternately optimised with the following objective functions:

(1) $$\begin{array}{cc} \min_{\Psi }\;\; & E_{s∼X_{S}}\left[L_{CE}\left(D\left(\phi_{S}\left(s;\Theta_{S}\right);\Psi \right),1\right)\right]+\\ \; & E_{t∼X_{T}}\left[L_{CE}\left(D\left(\phi_{T}\left(t;\Theta_{T}\right);\Psi \right),0\right)\right], \end{array}$$

(2) $$\min_{\Theta_{T}}\;\;E_{t∼X_{T}}\left[L_{CE}\left(D\left(\phi_{T}\left(t;\Theta_{T}\right);\Psi \right),1\right)\right],$$

where $L_{CE}$ is the cross-entropy loss, $\Theta_{S}$ is the set of the parameters of $\phi_{S}$ ($\Theta_{T}$ for $\phi_{T}$ respectively), and Ψ is the set of parameters for the discriminator.

As in [7,18], we emphasise that this feature alignment step using adversarial learning is unsupervised. In the next sections, we provide a description of the proposed variance-based regulariser and of the stopping criterion.

#### 3.2.1. Variance-Based Regularisation Preventing Posterior Collapse

During adversarial adaptation, the network may learn biased predictions on the target dataset. The worst scenario occurs when predictions clash to the same output, regardless of the input: this phenomenon is called *posterior collapse* [37]. To tackle this problem, we impose the network to increase the variance of the outputs of the regressor *R* when provided with the features generated by $\phi_{T}$. As such, we add a variance-based regulariser over the outputs of *R* (cf. Figure 2B), as follows:

(3) $$\max_{\Theta_{T}}\;\sigma_{{\hat{y}}_{t}}^{2}=\max_{\Theta_{T}}\;E\left[{\left({\hat{y}}_{t}-E\left[{\hat{y}}_{t}\right]\right)}^{2}\right],$$

where ${\hat{y}}_{t}$ is the output of the regressor when provided with the features generated by $\phi_{T}$, i.e., ${\hat{y}}_{t}=R\left(\phi_{T}\left(x_{t}\right)\right)$. This regulariser is applied during the optimisation of Equation (2).

Compared to other regularisers preventing the posterior collapse, such as Kullback-Leibler divergence used in [18], our variance-based regulariser has the advantage of not requiring labels from the source domain, i.e., it is *source-free*.

#### 3.2.2. Stopping Criterion

Finding a suitable stopping point during training mitigates overfitting. As, at this stage, we do not use labels from the target domain, we propose a novel stopping criterion that jointly exploits the Maximum Mean Discrepancy (MMD), as well as the GAN Global Optimality condition.

**MMD:** Let $X_{p}={\left\{x_{i}^{p}\right\}}_{i=1}^{M}$ and $X_{q}={\left\{x_{i}^{q}\right\}}_{i=1}^{N}$ be two sets of samples drawn i.i.d. from the distributions P and Q, H a universal reproducing kernel Hilbert space (RKHS), φ(·) the feature map associated with the kernel map $k\left(x^{p},x^{q}\right)=\langle \varphi \left(x^{p}\right),\varphi \left(x^{q}\right)\rangle$. We use the MMD, as proposed in [21,38], to compute an empirical estimation of the distance between P and Q and, therefore, to quantify the covariate shift. Thus, we compute the following:

(4) $${\mathrm{MMD}}^{2}\left(X_{p},X_{q}\right)={∥\frac{1}{M}\sum_{i=1}^{M}\varphi \left(x_{i}^{p}\right)-\frac{1}{N}\sum_{j=1}^{N}\varphi \left(x_{j}^{q}\right)∥}_{H}^{2}$$

We set $X_{p}=\phi_{S}\left(x_{s}\right)$ and $X_{q}=\phi_{T}\left(x_{t}\right)$, with $x_{s}\in X_{S}$, $x_{t}\in X_{T}$ and M=N=2048, i.e., as the size of the representations produced by the feature extractor. We use MMD as it is typically done with a validation loss: when it starts increasing, we stop the adversarial training.

**GAN Global Optimality Condition:** As proven in [22], the optimal discriminator $D^{*}\left(z\right)$ is reached when the discriminator is unable to differentiate between real and generated data. This happens when the generator distribution $p_{g}$ equals the data distribution $p_{data}$, i.e., $p_{g}=p_{data}$. If the adversarial adaptation is trained properly, in our case we expect that $P\left(\phi_{S}\left(x_{s}\right)\right)=P\left(\phi_{T}\left(x_{t}\right)\right)$. When this occurs, the output of the optimal discriminator $D^{*}\left(z\right)=\frac{1}{2}$, $\forall z\in \Phi =\Phi_{S}\cup \Phi_{T}$ ($\Phi_{S}$ and $\Phi_{T}$ are the feature spaces for the source and target dataset respectively—cf. Section 2.1). This means that, after a certain number of epochs, we will have $E_{z∼\Phi }\left[D(z)\right]=\frac{1}{2}$. Hence, when the function:

(5) $$\mathrm{GGO}\left(z\right)=E_{z∼\Phi }\left[D(z)\right]-\frac{1}{2}$$

is starting to increase, we can terminate the adversarial adaptation process.

Our results show that either Equation (4) or (5) may not always lead to a good stopping point. Therefore, we combine both as follows: during training, we observe both Equations (4) and (5) at each epoch and we save their best values. If neither of the two stopping criteria have improved for 10 epochs, then training is terminated. We demonstrate the effectiveness of our stopping criterion (together with the variance-based regulariser) in an ablation study in Section 4.4.

### 3.3. Fine-Tuning of the Regressor *R*

As discussed in Section 3.1, labels in $Y_{S}$ were normalised into [0,1]. In a real-world application, normalised predictions may be meaningless. To adjust the regressor to make predictions in the range of labels in the target set $Y_{T}$, we fine tune *R* with a handful of annotated images taken from the target dataset. Note that, up to this point, our approach has been unsupervised.

As displayed in Figure 2C, we put together the $\phi_{T}$, obtained from the previous step, and the regressor network *R*, obtained from the pretraining step. We fine-tune this model with a handful of annotated images taken from the target domain. We will show, in Section 4.5, that 10 annotated images are enough to successfully restore the predictions in $Y_{T}$. The resulting model is then used to make predictions in the target dataset.

### 3.4. Implementation Details

Prior training, images are rescaled to 320×320. Then, we perform a histogram normalisation as in [17]. Finally, we normalise input images in a range [−1,1] as in [18]. To prevent the overfitting, we employ data augmentation with flipping and colour jitter and initialise the feature extractor (ResNet-50) with the ImageNet weights.

During the fine-tuning step (cf. Figure 2C), we randomly select a handful of annotated images from the target dataset. In the Supplementary Materials, we report the list of hyperparameter utilised for training. The proposed approach was implemented with the framework Pytorch [39] and was trained on a GPU NVIDIA Quadro P5000.

Our code is available at https://github.com/MattiaLitrico/Semi-supervised-Domain-Adaptation-for-Holistic-Counting-under-Label-Gap (accessed on 20 September 2021).

## 4. Experimental Results

### 4.1. Datasets

In this section, we describe the datasets used to evaluate our semi-supervised DA approach. Overall, we test our method under three counting scenarios: (i) synthetic microscope images of cell; (ii) pedestrian; and (iii) plants.

**Figure 3:**
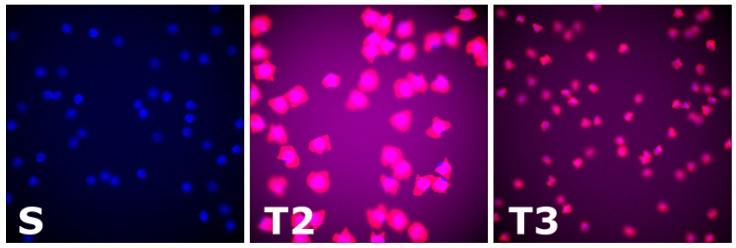
Samples of the cell datasets generated with [41]: *S* is the source dataset, and *T2* and *T3* are the target datasets. *S* and T2 were designed to exhibit covariate shift only, whereas *S* and T3 exhibit both covariate shift and label gap. (Best viewed in colour.)

**Cells:** As in [40], we adopted images of synthetic fluorescence microscopy of cells to benchmark our method. These images were generated using the framework proposed in [41]. However, the dataset used in [40] contains only 200 images (more information at https://www.robots.ox.ac.uk/~vgg/research/counting/index_org.html—accessed on 17 September 2021). Moreover, the dataset contains images generated from the same distribution, which is not suitable for domain adaptation purposes. Therefore, we generated 3 synthetic cell datasets, each containing 60,000 images of size 256×256 (cf. Figure 3). In particular, we generated:

- **S:** it contains images of blue cells with counting ranging in [20,50]. To generate these images, the option cytoplasm was disabled. We used this dataset as source domain. During training, we split the dataset as follows: 55% as training set, 20% as validation set (used for early stopping during pretraining), and 25% as test set.
- **T2:** it contains images of red cells (cytoplasm option enabled) with a counting ranging in [20,50] as well. This dataset is used as target domain to benchmark our approach in a scenario of covariate shift only.
- **T3:** similar to T2, but with a different cell counting ranging in [35,90]. To fit more cells in the same image, we generated smaller cells as in T2. This dataset exhibits both covariate and label gap.

**Pedestrian:** We used the publicly available UCSD [42] dataset containing videos of pedestrian in walkways acquired from stationary cameras. Specifically, the dataset contains the following videos of two different scenes (with different perspective):

- **Vidf**: it contains 4000 frames with people walking towards and away from the camera, with some amount of perspective distortion. These images have a pedestrian counting ranging in [11,45]. We used it as source domain for this experiment and the training/validation/testing sets are split as for the cell data.
- **Vidd:** it contains 4000 frames with pedestrian moving in parallel wrt the camera plane. The number of people appearing in the scene ranges in [0,15]. This dataset will serve as target domain for this experiment.

**Plants:** We also evaluated our method in the same plant biology context as in [18], namely leaf counting. Specifically, the following datasets are taken into consideration:

- CVPPP*: The CVPPP2017 dataset contains three subsets of *Arabidopsis thaliana* (named A1, A2, and A4), and tobacco (A3) images [43,44]. We used A1, A2, and A4 as source domain, i.e., excluding the tobacco plants (as in [18], we named this group of images CVPPP*). Overall, the CVPPP* dataset contains 964 images and a number of leaves ranging in [4,32]. For training, we split this dataset as in [17] to perform a 4-fold cross-validation for the pretraining step.
- **MM:** We use the RGB *Arabidopsis thaliana* images of the *Multi-Modal Imagery for Plant Phenotyping* [45] with 576 images and a leaf counting ranging in [5,12].
- **Komatsuna:** we use the Komatsuna, a Japanese plant, dataset [46], with 300 images and a leaf counting ranging in [2,8].

We refer to the adaptation from CVPPP* to MM as *intra-species*, as both datasets contain images of the same plant species. Differently, adapting from CPPP* to Komatsuna is referred to as *inter-species*. The leaf counting scenario is more challenging than the other two due to the limited dimension of datasets, especially for the inter-species case.

### 4.2. Evaluation Metrics

To evaluate the performance of our approach, we use the same metrics as in [17,18,47,48]. These evaluation metrics have been widely used in the CVPPP/CVPPA *Leaf Counting Challenges* (more information about the latest edition of this workshop is available at https://cvppa2021.github.io/—accessed on 17 September 2021). Let $\epsilon_{i}=y_{i}-round\left({\hat{y}}_{i}\right)$ be the prediction error (i.e., the difference between the ground truth *y* and the rounded algorithmic prediction $\hat{y}$), the evaluation metrics are defined as follows:

- *Absolute Difference in Count* [|DiC|]: $\frac{1}{N}\sum_{i=i}^{N}\left|\epsilon_{i}\right|$. This metrics is also known as *mean absolute error*;
- *Difference in Count* [DiC]: $\frac{1}{N}\sum_{i=i}^{N}\epsilon_{i}$;
- *Mean Squared Error* [MSE]: $\frac{1}{N}\sum_{i=i}^{N}\epsilon_{i}^{2}$;
- *Percentage Agreement* [%]: $\frac{1}{N}\sum_{i=i}^{N}1\left[\epsilon_{i}=0\right]$, where 1[n] is the indicator function. This metrics is similar to the accuracy used in classification.

### 4.3. Main Results

Here, we present the experimental results of the proposed semi-supervised DA approach for holistic counting of cells, pedestrians, and leaves in plants. We compare our approach with the one proposed in [18]. We also compare our approach with DANN [5], as it is another approach in literature that can be easily applied to holistic counting. Together with the DA results, we also report the *upper bound* (UB) and the *lower bound* (LB) results: in this context, UB is obtained by feeding the pretrained model (cf. Section 3.1) with the target dataset (e.g., no adaptation step); LB is obtained by training the feature extractor and regressor network directly on the target domain (fully supervised).

Section 4.4 shows the benefit of the variance-based regulariser (cf. Section 3.2.1) and the proposed combined stopping criterion (cf. Section 3.2.2). For all the results, we used 50 annotated images taken from the target dataset for the fine-tuning (cf. Section 3.3). Section 4.5 shows that, with just 10 annotated images, we obtained satisfactory results in all the three adopted datasets.

#### 4.3.1. Cell Counting Results

These experiments serve as a benchmark for our method, as T2 exhibits only covariate shift wrt *S*, whilst T3 exhibits both covariate shift and label gap. Overall, the experimental results are reported in Table 2.

**S → T2:** In the presence of covariate shift only, the approach in [18] outperforms ours. Despite that, our results show an MSE <1, i.e., our method is approx. ±1 cell off.

**S → T3:** As stated above, T3 differs from S not only in appearance but also in the total number of cells per image. In this situation, our approach drastically outperforms the others across the board, reducing the MSE from 759 to 5.62. By observing the DiC, it can be seen that both DANN and the approach in [18] always underestimates the number of cells. As T3 contains many images with a cell counting above the label range in S, we argue that this occurs because those approaches have never seen samples with a count over 50 cells and, thus, cannot predict numbers outside the source label range.

These benchmark experiments demonstrate that our method well aligns the two datasets under label gap. Next, we present the DA results on two publicly available datasets taken from real-world applications that also demonstrates the ability of our approach to face the label gap.

#### 4.3.2. Pedestrian Counting Results

We use the UCSD dataset [42] for the pedestrian counting task. Specifically, we use the *Vidf* scene as source domain and the *Vidd* scene as target. The domain shift is due to different: (i) camera perspectives; (ii) locations. Moreover, the label ranges are highly different between the two datasets, exhibiting label gap. As shown in Table 3, also in this experiment our approach drastically outperforms the others.

**Figure 4:**
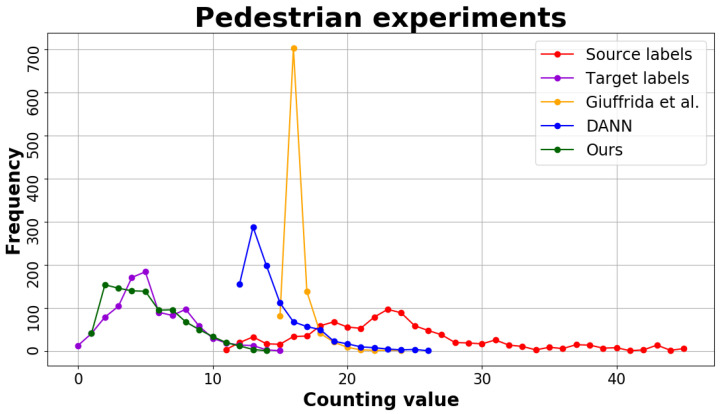
Histogram visualisation of counting frequencies in Pedestrian experiments. (Best viewed in colour.)

Figure 4 shows the performance of our method against the others: it can be seen that the predictions of our method (green line) in the target domain are very similar to the ground-truth (purple line). On the contrary, the predictions made by [18] are condensed in the range [15,20] (yellow). Also DANN [5] struggles to correctly predict the number of pedestrian in the target domain (blue line). This confirms our hypothesis that these methods cannot make predictions outside the range of the source dataset (red line).

Therefore, this experiment demonstrates that our approach is able to perform DA also in a real-world application. The next experiment exhibits an extra challenge, as source and target datasets have a limited number of images.

#### 4.3.3. Leaf Counting Results

Similarly as in Section 4.3.2, we assess the performance of our method on another real-world scenario applied to plant biology. For the following experiments, we used the CVPPP* dataset as source domain. The first experiment considers the MM [45] dataset as target and shows the ability of our approach to perform domain adaptation in the *intra-species* scenario, as both source and target domains include images of the same plant species. In the second test, we show the ability of our approach to also successfully perform domain adaptation in the *inter-species* scenario. Both experimental results are shown in Table 4.

Overall, it can be noted that our proposed method outperforms the others also in this set of experiments, lowering the MSE < 1 and increasing the percentage agreement (on average) by ∼20%. Furthermore, the obtained results are very close to the lower bound in both intra- and inter-species experiments.

**Figure 5:**
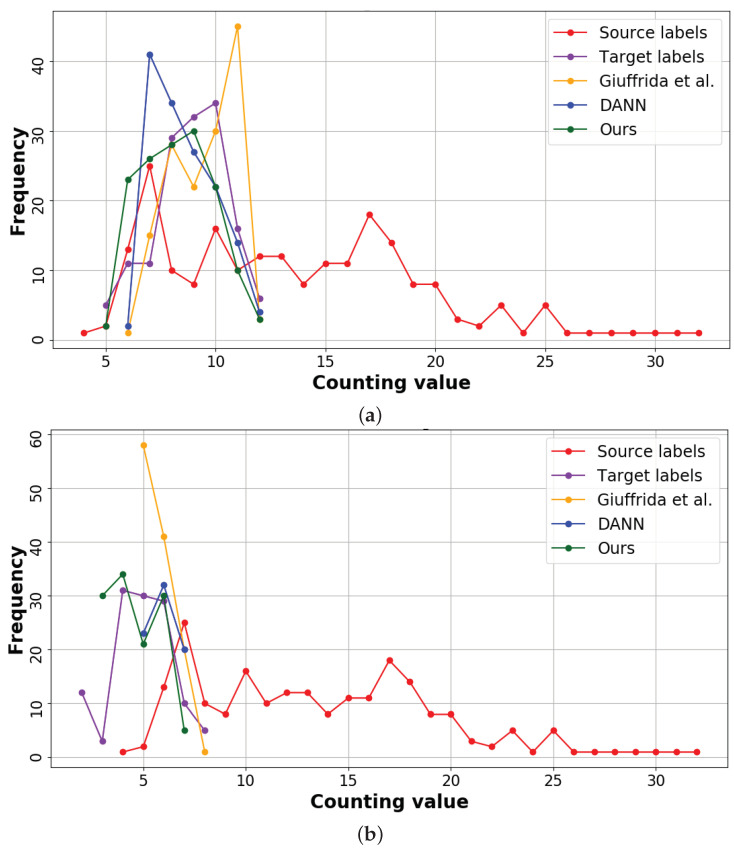
Visual representation of the leaf counting results. (**a**) *Intra-species:* CVPPP* [43,44]→MM [45]. (**b**) *Inter-species:* CVPPP* [43,44]→Komatsuna [46].

As we displayed for the pedestrian dataset (cf. Section 4.3.2), Figure 5 visualises the counting values (and their frequencies) for the leaf counting datasets: for both intra- (cf. Figure 5a) and inter-species (cf. Figure 5b), our approach well approximates the target label distribution.

### 4.4. Ablation Study

To assess the effectiveness of the stopping criterion (cf. Section 3.2.2), as well as of the variance-based regulariser (cf. Section 3.2.1), we perform an ablation study removing, alternately, each of these components using the cell and the UCSD datasets(cf. Section 4.3.1 and Section 4.3.2). Results, shown in Table 5, prove that the combination of both stopping criterion and regulariser achieves the best performance. It can be noted that either the use of Equation (5) or (4) does not always lead to a good stopping point, as the experiments with the cell dataset demonstrate, compared to the ones with the pedestrian dataset.

Overall, this study shows that each component of our method contributes to mitigate overfitting. Furthermore, it also demonstrates the effectiveness of the proposed regulariser to prevent posterior collapse.

### 4.5. Fine-Tuning Performance Analysis

In the third training step of our approach (cf. Section 3.3), we fine-tune the regressor with a reduced number of random annotated images sampled from $X_{T}$. We perform this last semi-supervised step to remap the predictions in target dataset from [0,1] to $Y_{T}=\left[a^{\prime },b^{\prime }\right]$. In Section 4.3, we show the achieved results using 50 annotated samples from the target domain $X_{T}$.

**Figure 6:**
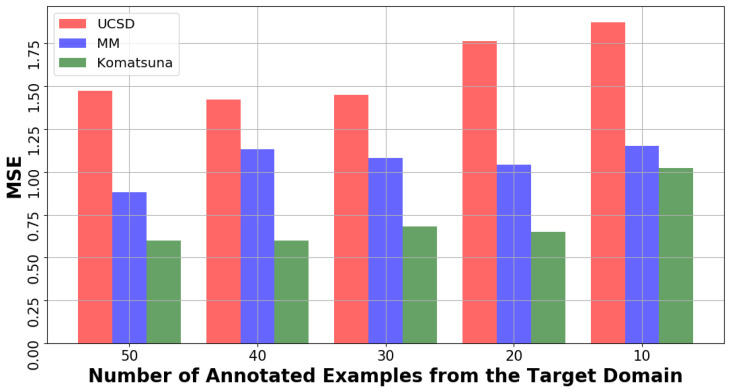
MSE variation during fine-tuning (cf. Section 3.3) wrt a decreasing number of annotated samples from the target dataset.

Here, we want to analyse the performance of our method with a decreasing number of annotated examples from $X_{T}$. Figure 6 shows the variation of MSE in pedestrian and plant experimental setups. Overall, the performance remains very stable and satisfactory, even with only 10 samples, as the MSE is always <2. From a practical perspective, the annotation of 10 random images taken from the target domain is a rather tractable task.

## 5. Conclusions

In this paper, we proposed a semi-supervised domain adaptation (DA) approach for the holistic counting task, where a model predicts a continuous value y∈R. The proposed approach was devised to jointly tackle covariate shift and label gap. We employed adversarial training to reduce the covariate shift, and we normalised the label range (in the source domain) to tackle the label gap. As a consequence, our method can be used under *closed set*, *open set* and *partial* DA.

To reduce overfitting, we proposed a stopping criterion that monitors both *GAN Global Optimality* (GGO) and *Maximum Mean Discrepancy* (MMD) conditions to determine a good stopping point and, thus, to learn a better feature representation. Furthermore, we proposed a variance-based regulariser to prevent posterior collapse. The effectiveness of each component in our method was demonstrated with an ablation study.

Lastly, we used a handful of annotated images from the target dataset to restore the original label range and we demonstrated that as fewer as 10 annotated images are enough to obtain stable and satisfactory results.

Overall, our method outperformed the state-of-the-art across the board under three different scenarios: cell, pedestrian, and leaf counting. Furthermore, our method also demonstrated to successfully perform domain adaptation also when limited datasets are provided, as shown in the leaf counting experiments. Particularly in this case, our method narrowed the MSE toward the lower bound performance.

The main limitation of our approach is the semi-supervised training, although it requires a handful of annotated images in the testing set. Future works should focus on making the training fully unsupervised with the help of additional tasks. Furthermore, the use of an alternative adversarial loss (e.g., least squares [36] or Wasserstein [49,50]) is another avenue of improvement, as it may yield better results.

## Figures and Tables

**Table 1:** Architectures details of the Regressor and Discriminator. We set α=0.01 for the LeakyReLU.

Layer	Input Size	Output Size	Activation
*Regressor*			
Dense	2048	1024	ReLU
Dense	1024	512	ReLU
Dense	512	1	Linear
*Discriminator*			
Dense	2048	1024	LeakyReLU
Dense	1024	512	LeakyReLU
Dense	512	1	Sigmoid

**Table 2:** Cell counting domain adaptation results for S→T2 and S→T3 experiments. Together with the DA results, we also report the *upper bound* (UB)—testing on the target dataset without the adversarial adaptation step—and the *lower bound*—supervised learning on the target dataset. Main results are highlighted in grey. For DiC and |DiC| we report *mean*(*std*).

Method	$X_{T}$	|DiC| ↓	DiC ↓	MSE ↓	% ↑
UB	T2	24.39(7.40)	24.39(7.40)	650	0.0
DANN [5]	T2	3.11(2.53)	1.16(3.84)	16.12	10.8
Giuffrida et al. [18]	T2	0.16(0.38)	0.03(0.41)	0.17	83.7
Ours	T2	0.53(0.58)	0.03(0.79)	0.63	50.9
LB	T2	0.01(0.10)	−0.01(0.10)	0.01	99.1
UB	T3	59.12(14.54)	59.12(14.54)	3708	0.0
DANN [5]	T3	20.79(11.01)	20.79(11.01)	553	0.0
Giuffrida et al. [18]	T3	26.33(8.14)	26.33(8.14)	759	0.0
**Ours**	T3	1.84(1.48)	0.04(2.37)	5.62	17.0
LB	T3	0.36(0.49)	−0.01(0.61)	0.37	64.3

**Table 3:** Pedestrian counting domain adaptation experiments results using the UCSD dataset. The Vidf scene is used as source domain whereas the Vidd scene is used as target domain.

Method	|DiC| ↓	DiC ↓	MSE ↓	% ↑
UB	2.54(1.68)	2.50(1.73)	9.28	8.60
DANN [5]	9.58(1.47)	−9.58(1.47)	94	0.0
Giuffrida et al. [18]	11.22(2.47)	−11.22(2.47)	132	0.0
**Ours**	0.89(0.82)	−0.12(1.21)	1.47	34.5
LB	0.15(0.37)	0.01(0.40)	0.16	84.8

**Table 4:** Leaf counting DA results for the CVPPP* → MM (*intra-species*), and CVPPP* → Komatsuna (*inter-species*) scenarios.

Method	|DiC| ↓	DiC ↓	MSE ↓	% ↑
*Intra-species:* CVPPP* → MM				
UB	1.76(0.99)	1.47(1.39)	4.11	8.33
DANN [5]	0.85(0.84)	−0.15(1.18)	1.43	37.5
Giuffrida et al. [18]	1.18(0.98)	−0.39(1.49)	2.36	26.0
**Ours**	0.67(0.65)	0.15(0.92)	0.88	43.1
LB	0.54(0.53)	0.24(0.72)	0.59	47.2
*Inter-species:* CVPPP* → Komatsuna				
UB	4.82(1.38)	4.82(1.38)	25.19	0.0
DANN [5]	1.72(1.02)	−1.64(1.15)	4.01	10.6
Giuffrida et al. [18]	1.04(0.87)	−0.78(1.12)	1.84	26.0
**Ours**	0.54(0.56)	−0.15(0.76)	0.60	49.2
LB	0.34(0.31)	−0.21(0.68)	0.47	54.4

**Table 5:** Ablation study on cell and pedestrian datasets. **GGO** indicates the GAN Global Optimality Condition; **MMD** is the Maximum Mean Discrepancy; σ${}^{2}$ is the variance-based regulariser.

GGO	MMD	σ ${}^{2}$	MSE ↓		% ↑	
			*Cell*	*UCSD*	*Cell*	*UCSD*
✓	-	✓	8.61	1.47	14.9	33.4
-	✓	✓	6.66	2.23	15.8	25.7
✓	✓	-	6.38	1.51	16.6	34.4
✓	✓	✓	5.62	1.47	17.0	34.6

## Data Availability

Not applicable.

## Supplementary Materials

The following are available online at https://www.mdpi.com/article/10.3390/jimaging7100198/s1, Table S1: Notation adopted in the paper, Table S2: Hyperparameters used in the experiments, Figure S1: Fine-tuning performance comparisons of our method against [18] on all the real-world used datasets.
