# Supplementary material for: Semi-Supervised Domain Adaptation for Holistic Counting under Label Gap
Source: J Imaging. 2021 Sep 29;7(10):198. doi: 10.3390/jimaging7100198 (PMC8541592; doi:10.3390/jimaging7100198)
Supplement: Supplementary file 1 [file jimaging-07-00198-s001.zip › jimaging-1377201_SI.pdf]

Article

Semi-Supervised Domain Adaptation for Holistic Counting under Label Gap

Mattia Litrico [1,†], Sebastiano Battiato [2], Sotirios Tsaftaris[3], and Mario Valerio Giuffrida [4,*]

1 Department of Mathematics and Computer Science, University of Catania, Italy; mattia.litrico@studium.unict.it
2 Department of Mathematics and Computer Science, University of Catania, Italy; battiato@dmi.unict.it
3 School of Engineering, University of Edinburgh, UK; s.tsaftaris@ed.ac.uk
4 School of Computing, Edinburgh Napier University, UK; v.giuffrida@napier.ac.uk
* Correspondence: v.giuffrida@napier.ac.uk; Tel.: +44 131 455 2744
† This work was undertaken while visiting Edinburgh Napier University

Variable	Notation
Source domain	$\mathcal{X}_S$
Target domain	$\mathcal{X}_T$
Image in the source domain	x_s
Image in the target domain	x_t
Source labels	$\mathcal{Y}_S$
Target labels	$\mathcal{Y}_T$
Marginal source label distribution	$\mathcal{D}_S^{\mathcal{Y}}$
Marginal target label distribution	$\mathcal{D}_T^{\mathcal{Y}}$
Feature extractor	$\phi()$
Probability distribution	$\mathcal{P}()$
Generator	G
Discriminator	D
Regressor	R
Generator parameters	Θ
Discriminator parameters	Ψ

Table S1: Notation adopted in the paper.

Fine-tuning Comparisons against Giuffrida et al. [1]

As our approach includes a semi-supervised training step, the following question arises: *would the method in [1] benefit from a handful of annotations taken from the target dataset?* Therefore, we trained the method in [1] with the pedestrian [2] and leaf counting [3–6] datasets (as described in Section 4.3.2 and 4.3.3 in the main paper) and then we fine-tuned the regressor with a ranging numbers of labelled images taken from the target datasets, as we did for our method in Section 4.5 in the main paper. We compared the results obtained with the fine-tuned regressor of [1] with ours and we show the results in Figure S1.

Overall, the approach in [1] does benefit from this fine-tuning, reducing the MSE on all the 3 experimental setups, compared with the results obtained without fine-tuning, (c.f. Tables 3 and 4 in the main paper). However, the method in [1] never outperforms ours (only for the intra-species case the MSE is closer to ours, although still higher – c.f. Figure S1 (b)), demonstrating that the additional supervision provided to their method is still not enough to address the label gap problem, especially for the pedestrian (c.f. Figure S1 (a)) and inter-species (c.f. Figure S1 (c)) cases.

Hyperparameter	Value
Pretraining Step	
Batch Size	64
Optimiser	*Adam*
Learning Rate	0.0001
Weight Decay	0.01
Adversarial Adaptation Step	
Batch Size	32
Optimiser Generator	*Adam*
Optimiser Discriminator	*SGD*
Learning Rate	0.0001
Weight Decay	0.01
Variance Regulariser	0.01
Regressor Fine-tuning Step	
Batch Size	64
Optimiser	*Adam*
Learning Rate	0.0001

Table S2: Hyperparameters used in the experiments.

References

1. Giuffrida, M.V.; Dobrescu, A.; Doerner, P.; Tsaftaris, S.A. Leaf Counting Without Annotations Using Adversarial Unsupervised Domain Adaptation. 2019 IEEE/CVF Conference on Computer Vision and Pattern Recognition Workshops (CVPRW), 2019, pp. 2590–2599. doi: 10.1109/CVPRW.2019.00315.
2. Chan, A.B.; Vasconcelos, N. Counting People With Low-Level Features and Bayesian Regression. *IEEE Transactions on Image Processing* **2012**, *21*, 2160–2177. doi:10.1109/TIP.2011.2172800.
3. Bell, J.; Dee, H.M. Aberystwyth Leaf Evaluation Dataset, 2016. doi:10.5281/zenodo.168158.
4. Cruz, J.; Yin, X.; Liu, X.; Imran, S.; Morris, D.; Kramer, D.; Chen, J. Multi-modality Imagery Database for Plant Phenotyping. *Machine Vision and Applications* **2016**, *27*, 735–749.
5. Minervini, M.; Fischbach, A.; Scharr, H.; Tsaftaris, S.A. Finely-grained annotated datasets for image-based plant phenotyping. *Pattern Recognition Letters* **2016**, *81*, 80–89. doi: https://doi.org/10.1016/j.patrec.2015.10.013.
6. Uchiyama, H.; Sakurai, S.; Mishima, M.; Arita, D.; Okayasu, T.; Shimada, A.; Taniguchi, R. An Easy-to-Setup 3D Phenotyping Platform for KOMATSUNA Dataset. 2017 IEEE International Conference on Computer Vision Workshops (ICCVW), 2017, pp. 2038–2045. doi:10.1109/ICCVW.2017.239.

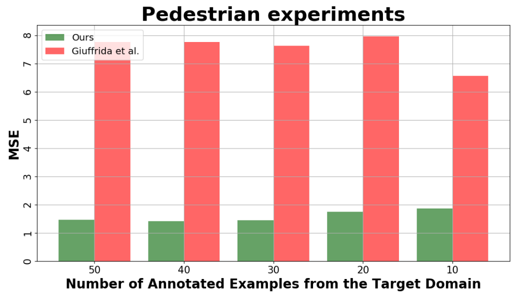

(a)

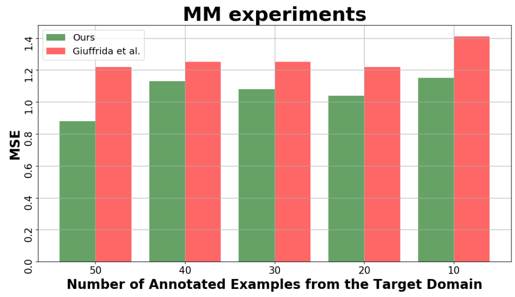

(b)

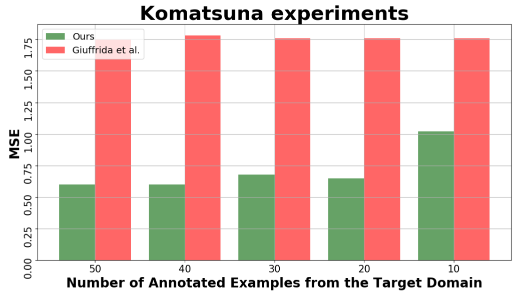

(c)

Figure S1. Fine-tuning performance comparisons of our method against [1] on all the real-world datasets utilised in this work (c.f. Section 4.3.2 and 4.3.3 in the main paper). We report the MSE wrt a variable number of annotated images taken from the target domains. (a) Pedestrian dataset [2] fine-tuning experiment; (b) Intra-species leaf counting fine-tuning experiment; (c) Inter-species leaf counting fine-tuning experiment.
